# Sperm use economy of honeybee (*Apis mellifera*) queens

**DOI:** 10.1002/ece3.2075

**Published:** 2016-03-22

**Authors:** Boris Baer, Jason Collins, Kristiina Maalaps, Susanne P. A. den Boer

**Affiliations:** ^1^ Centre for Integrative Bee Research (CIBER)ARC Centre of Excellence in Plant Energy BiologyThe University of Western AustraliaMCS Building M3106009Western AustraliaCrawleyAustralia; ^2^ Business SchoolThe University of Western AustraliaBuilding M2526009Western AustraliaCrawleyAustralia; ^3^ Department of BiologyCentre for Social EvolutionUniversity of CopenhagenUniversitetsparken 152100CopenhagenDenmark

**Keywords:** DAPI, egg fertilization, fertility, sperm storage, spermatheca

## Abstract

The queens of eusocial ants, bees, and wasps only mate during a very brief period early in life to acquire and store a lifetime supply of sperm. As sperm cannot be replenished, queens have to be highly economic when using stored sperm to fertilize eggs, especially in species with large and long‐lived colonies. However, queen fertility has not been studied in detail, so that we have little understanding of how economic sperm use is in different species, and whether queens are able to influence their sperm use. This is surprising given that sperm use is a key factor of eusocial life, as it determines the fecundity and longevity of queens and therefore colony fitness. We quantified the number of sperm that honeybee (*Apis mellifera*) queens use to fertilize eggs. We examined sperm use in naturally mated queens of different ages and in queens artificially inseminated with different volumes of semen. We found that queens are remarkably efficient and only use a median of 2 sperm per egg fertilization, with decreasing sperm use in older queens. The number of sperm in storage was always a significant predictor for the number of sperm used per fertilization, indicating that queens use a constant ratio of spermathecal fluid relative to total spermathecal volume of 2.364 × 10^−6^ to fertilize eggs. This allowed us to calculate a lifetime fecundity for honeybee queens of around 1,500,000 fertilized eggs. Our data provide the first empirical evidence that honeybee queens do not manipulate sperm use, and fertilization failures in worker‐destined eggs are therefore honest signals that workers can use to time queen replacement, which is crucial for colony performance and fitness.

## Introduction

The storage of sperm by females prior to egg fertilization is a phylogenetically widespread phenomenon and occurs in a range of animals such as insects, arachnids, reptiles, birds, or mammals (Birkhead and Møller 1993; Neubaum and Wolfner 1999; Simmons 2001; Holt and Lloyd 2010; Orr and Zuk 2012). It is often facilitated by the presence of specialized morphological structures in the females' sexual tract such as sperm tubes or spermathecae (Walker 1980; Birkhead and Møller 1993; Eberhard 1996; Neubaum and Wolfner 1999). Sperm storage offers a number of advantages. It allows females to fertilize eggs over prolonged periods of time, thereby securing fertility in the absence of males, and reducing the need for costly additional matings. Furthermore, the separation of mating and egg fertilization offers opportunity for paternity manipulations, for example, through postcopulatory sexual selection favoring the most competitive (sperm competition; e.g., Parker 1970; Simmons 2001) or most preferred males (cryptic female choice; e.g., Eberhard 1996; Arnqvist 2014). However, how much control females have over the processes occurring during the storage and use of sperm has not been quantified in any great detail.

The queens of the social Hymenoptera (the eusocial bees, ants, and wasps) have driven sperm storage and use to spectacular extremes. In these species, queens never remate after an initial mating episode early in their lives where they acquire a lifetime supply of sperm. In some species, queens can survive for years to decades and head colonies with millions of workers (Weber 1972; Pamilo 1991; Keller and Genoud 1997; Keller 1998; Baer 2005). As a consequence, males of some social insect species produce exceptionally large ejaculates of high quality (Fjerdingstad and Boomsma 1997; Schlüns et al. 2003; den Boer et al. 2008; den Boer et al. 2009a) and queens are able to efficiently keep stored sperm alive (Boomsma et al. 2005) and use them economically during egg fertilization. Hymenopteran queens have some control over sperm use, because they can switch between the production of fertilized eggs developing into female offspring (workers and queens), and nonfertilized eggs developing into males (e.g., Ratnieks and Keller 1998; Aron 2012). Despite the recognition that sperm use is a key determinant of sex ratios, colony size, and longevity (Baer 2016), few studies have quantified this key aspect of eusocial life, in particular whether queens are able to manipulate their sperm economy. Several studies attempted to estimate average sperm use in ants, wasps, and bees, but these analyses relied on indirect and correlative approaches (Harbo 1979; Tschinkel and Porter 1988; Stein et al. 1996). Counting the number of sperm in spermathecae of queens at different ages, and estimating the approximate numbers of eggs queens had laid at that point, allowed calculations of sperm usage by queens, but these values were highly variable ranging from 3 to 100 sperm per fertilization. To overcome the limitations of these early approaches, we previously developed a novel method to directly count the number of sperm on freshly laid eggs for the leafcutter ant *A. colombica* (den Boer et al. 2009b). This work confirmed that queens use very few sperm per fertilization, but also showed that sperm use of queens increases with age.

Here, we chose to study sperm economy in the honeybee *Apis mellifera*, because the life history of this species implies strong selection on sperm use. Honeybee queens are known for their exceptionally high mating frequencies (Koeniger and Koeniger 2000; Tarpy et al. 2004). They only store around 3–5% of the sperm they acquired during copulations (Baer 2005), but are nevertheless extremely fertile and able to produce up to 1.7 million fertilized eggs over a life span of up to 8 years (Bozina 1961). These data imply that honeybee queens are under strong selection to minimize the numbers of sperm used per fertilization, but previous estimations of sperm usage in honeybees were quite variable and ranged from 4 to 100 sperm per egg (Bresslau 1905; Adam 1912; Ruttner 1975; Harbo 1979; Yu and Omholt 1999).

Honeybees differ from other social insects in that workers can kill a resident mother queen and replace her with one of her daughters, which is known as supersedure (Butler 1957; Winston 1991). The replacement of the queen should be initiated as soon as a queen's fertility declines and workers can, in principle, assess queen fertility by monitoring her egg laying frequency and/or the success rate of egg fertilization. The latter is phenotypically expressed in brood patterns, because unfertilized eggs develop into male brood. Queens are normally extremely accurate in fertilizing eggs, and unfertilized (male) eggs are rarely found in smaller worker‐destined brood cells (Ratnieks and Keller 1998). Workers could therefore use fertilization failure of worker‐destined eggs as a reliable signal to initiate queen supersedure. However, if honeybee queens are able to manipulate their sperm use, they are expected to (1) minimize sperm use when young and (2) delay their replacement later in life by increasing the number of sperm used per fertilization as they get older, thereby compensating for fertilization failures that could result from senescence of their reproductive tract and/or sperm (den Boer et al. 2009b). Consequently, knowledge about a queen's influence on sperm usage provides insights into potential reproductive conflicts that occur in these societies and how they have been resolved over evolutionary time frames.

We quantified sperm usage in honeybees by counting the number of sperm on freshly laid eggs. This allowed us to test a number of hypotheses: First, we predicted that young honeybee queens are extremely economic in their sperm use and quantified this in 22 naturally mated queens. Second, we predicted that – similar to leafcutter ant queens – older queens increase sperm use to delay their supersedure. Sperm use should therefore be independent of the number of sperm stored in the spermatheca, and we tested this idea by artificially inseminating virgin queens with different volumes of semen and quantifying their sperm use in the first eggs laid. Finally, we developed a mathematical model to describe sperm use in honeybees and tested whether it can accurately predict our empirical data.

## Material and Methods

### Egg collection and sperm counting

All honeybee queens used for experiments were kept in an apiary at the Centre for Integrative Bee Research (CIBER) at the University of Western Australia. The Mediterranean climate in Perth allows honeybees to remain active all year round, which had two important consequences. First, we were able to continuously quantify sperm usage of queens, and collected data between March 2012 and February 2013. Second, such continuous egg laying limits the typical life span of queens to up to 3 years. As we collected data from queens of up to 28 months of age, we were able to obtain sperm use data that covered most of an average queen's life span. Prior to the experimental work, we checked that queens displayed normal egg laying behavior, indicated by the presence of eggs and larvae in colonies and the absence of male brood in worker cells. Fertilization failure of honeybee queens (who are then referred to as drone layers) has three distinct phenotypes that can be observed when examining colonies: first, the appearance of empty cells, as workers recognize nonfertilized eggs and remove them (known as patchy brood); second, the formation of deformed brood cells, because the larger sized males eventually outgrow the smaller worker cells; third, the appearance of dwarf males as male larvae are not able to grow to their full size when reared in worker cells. We monitored colonies for the appearance of these phenotypical signs of fertilization failures of queens but did not detect any of these.

To sample freshly laid eggs, we collected queens from their colonies and caged them on an empty worker brood frame using a confinement made from commercially available plastic queen excluders. This setup restricted the queen's ability to move around to a defined area of approximately 16 cm × 16 cm on one side of a worker brood frame, but allowed workers to freely enter and leave the area. Frames were placed back into their hives, and the caged queens were subsequently allowed to lay eggs for 1.5–3 h. We then recollected the frames from the colonies and released the queens back into their colonies. In the laboratory, we collected eggs from individual cells with a needle and transferred them to a microscope slide that we kept in a container with moist tissue to avoid desiccation of eggs. To count the number of sperm on each egg, we stained eggs with 5 *μ*L of DAPI working solution, which we initially prepared by mixing 2 mL of DAPI stock solution (2 mg DAPI dissolved in 1 mL dimethylsulfoxide) with 1 mL of 0.1 mol/L NaPO4, pH 7.0. We then placed a cover slide over each egg, causing them to break open so that the DAPI stain could equally disperse and effectively stain the DNA of sperm heads. All samples were examined using a fluorescence microscope (Axio Imager A1, Zeiss, Oberkochen, Germany) and the number of sperm heads counted on each egg, using between 27 and 60 eggs per queen in total. Because queens hardly ever lay unfertilized eggs in worker cells (Ratnieks and Keller 1998), and because we could confirm the absence of male brood in worker combs, we assumed that all eggs sampled were fertilized. We therefore added one additional sperm to all counts to include the sperm cell that had fertilized the egg, similar to the approaches we used earlier in leafcutter ants (den Boer et al. 2009b).

### Sperm use and number of sperm stored in queens of different ages

To quantify the effect of queen age on sperm use, we examined 22 naturally mated queens that were between 1 and 28 months old. The age range used for this experiment included queens that had just started to lay eggs, shortly after mating and old queens near the end of their life and therefore close to their maximal reproductive capacity. For 8 of the 22 queens examined, we were able to remeasure sperm use over several months. We immediately killed queens after egg counting was completed, dissected their spermatheca, and counted the number of sperm stored. Because not all queens survived to the end of the experiment, we obtained sperm counts for 14 of the 22 queens.

To count the number of stored sperm, we dissected the spermatheca from a queen and transferred it to a lid of an Eppendorf tube containing 10 *μ*L of Hayes solution (0.15 mol/L NaCl, 1.80 mmol/L CaCl_2_, 2.68 mmol/L KCl, 1.19 mmol/L NaHCO_3_, adjusted to pH 8.7 using NaOH). The spermatheca was gently ruptured, and outflowing sperm was mixed with 190 *μ*L of Hayes in an Eppendorf tube by gently turning it 10 times. We used 5 *μ*L of this sperm stock solution and added 295 *μ*L of distilled water, briefly vortexed the sample, and placed four droplets of 1 *μ*L each on a microscope slide to let them air‐dry. To count the number of sperm, we added 2 *μ*L of DAPI working solution to each droplet, covered them with a cover slide, and inspected each sample using a fluorescence microscope. To obtain the number of sperm in the spermatheca, we multiplied the number of sperm counted in each 1‐*μ*L droplet by 12,000. For statistical analyses, we used the mean number of sperm counted for the four droplets.

### Sperm usage in queens of the same age

To test whether or not the number of sperm used per fertilization depends on the number of sperm present in the spermatheca, we quantified sperm use patterns of queens of similar age that differed in the amount of sperm present in the spermatheca. To do this, we bred virgin queens using standard apiarists methods (Graham 1992). We collected 3‐day‐old larvae from worker brood comb and transferred them to commercially available queen cells before placing them in a queenless foster hive. After 10 days, queen cells were recollected and each cell was introduced into a nucleus hive where queens hatched 2–3 days later. When queens reached an age of 7 days, we artificially inseminated seven virgin queens with 3 *μ*L and seven virgin queens with 12 *μ*L of semen according to a previously developed protocol (Schley 1987). Semen used for artificial inseminations originated from males that we bred in several colonies in the same beeyard. Sexually mature males were collected at the entrance of their hives while returning from nuptial flights. For the collection of semen, males were allowed to fly in a flight cage in the laboratory for a few minutes before they were anaesthetized with chloroform, which initiated the ejaculation process in most males. Complete eversion of the endophallus was subsequently evoked by gently squeezing the male's abdomen between two fingers. We collected outflowing semen with a glass capillary and inseminated queens using a standard Schley artificial insemination apparatus (Schley 1987). After insemination, queens were placed back in queen cages in separate queenless colonies. They were left in these queen cages for 3–5 days before being released into the nucleus hive to ensure worker bees would be accustomed to a queen's scent and accept her. Queens were left undisturbed for at least 2 weeks before their eggs were collected as described above. As soon as 25 eggs were analyzed, queens were killed and the number of stored sperm was determined as described above.

### Statistics

All statistical analyses were carried out using IBM SPSS, version 22 for Windows (IBM Corp., Armonk, NY, USA). Because sperm use per egg was heavily skewed and could not be fitted to parametric distributions, we initially used nonparametric statistics to test for differences in sperm use between queens. For subsequent parametric analyses of variation in sperm use between queens, we used the median sperm usage for each queen instead of the mean.

Queen age was defined by rounding up to nearest month after hatching, and an age class was included in the statistical analyses if at least five eggs had become available to quantify sperm use. For 8 of the 22 queens, sperm use measurements became available for several ages, and we used median sperm use per age for statistical analyses. To examine the overall effect of age on sperm use, we only used data of the first age class measured per queen. However, to investigate whether the number of sperm in storage influenced the number of sperm used per fertilization, we used the latest sperm use data, that is, those collected directly prior to sacrificing queens to determine the number of sperm in their spermathecae.

## Results

### Sperm usage in naturally mated queens of different ages

**Figure 1:**
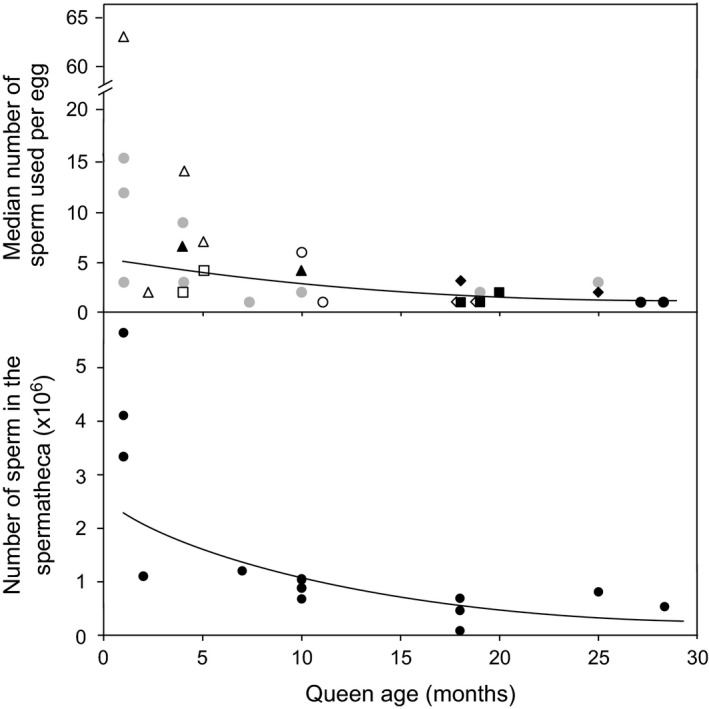
Sperm used and the number of sperm stored in the spermatheca plotted as a function of queen age, using naturally mated queens. (A) The relationship between queen age and sperm use during egg fertilization in 22 queens. Gray dots are used for queens that were examined in a single month. Black symbols are used for queens that were examined for longer periods, and the same symbols represent the same queens. A regression line of ln(*X*_*t*_) = 1.67 − 0.06631*t* was calculated using only one age class per queen (the youngest age class in queens with multiple age classes), excluding queen 13 (open triangles). Please note that the *y*‐axis is not continuous. (B) The relationship between queen age and the number of sperm stored in the spermatheca in 14 queens. The regression line shown is ln(*N*_*t*_)* *= 14.72 − 0.0835*t*.

We found that the median sperm use overall was two sperm per egg, but sperm use differed significantly between queens (Kruskal–Wallis test, *H* = 521.342, df = 21, *P* < 0.001; Fig. 1A). A closer inspection of the data revealed that a single queen was characterized by remarkable variation in her sperm use (see open triangles in Fig. 1A), ranging from 1 to 428 (median = 15) sperm used per fertilization. Because the sperm use of this queen differed substantially from the other 21 queens we investigated, we removed this queen from subsequent analyses. However, even after removal of this queen from the data set, sperm use still differed significantly between queens (Kruskal–Wallis test, *H* = 482.631, df = 20, *P* < 0.001).

When we analyzed the effect of queen age on sperm use, we found that – contrary to our expectation – older queens used significantly fewer sperm compared to younger queens (*F*_1,19_ = 14.096, *P* = 0.001; Fig. 1A). When we separately analyzed the data set for queens that were younger or older than 12 months, we found that median sperm use was higher in young queens (5.42 ± 1.32 SE) compared to old ones (1.56 ± 0.29 SE, Welch *t*‐test, *t* = 3.085, df = 14.961, *P* = 0.008). Even though sperm use generally declined in older queens, this was not consistent for those queens where we were able to measure sperm use multiple times (paired *t*‐test, *t* = 0.887, df = 6, *P* = 0.409; Fig. 1A). We also found a significant difference in the variation of median sperm use between age groups; older queens were more consistent in their sperm use compared to younger queens (*F*‐test of equality of variances; *F*_14,13_ = 31.127, *P* < 0.001), and there was no significant difference in the sperm usage between the older queens (Kruskal–Wallis, *H* = 8.000, df = 8, *P* = 0.433).

The number of sperm stored in the naturally mated queens was highly variable and ranged from 57,000 to 5,652,000. As expected, we found that the number of stored sperm was highest in 1‐month‐old queens (4,365,000 ± 680,903, mean ± SEM) and significantly decreased in older queens (*F*_1,12_ = 34.637, *P* < 0.001) (Fig. 1B).

### Sperm use in queens artificially inseminated with different amounts of ejaculate

**Figure 2:**
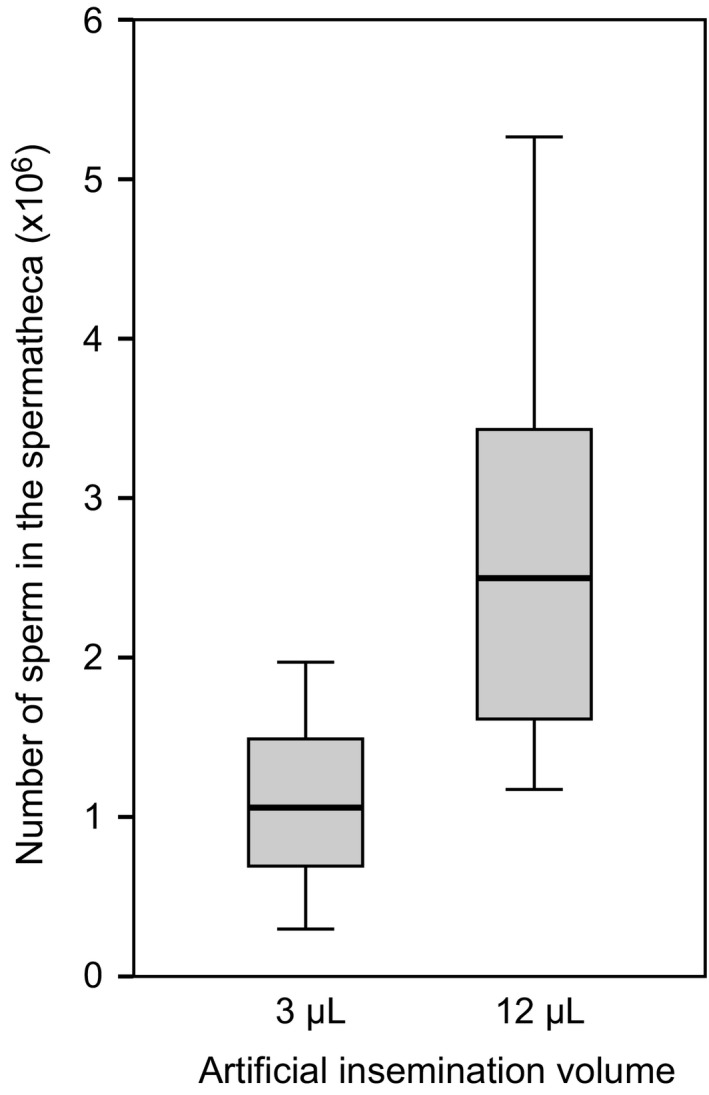
Boxplots depicting the number of sperm stored in the spermatheca after insemination with either 3 *μ*L or 12 *μ*L of semen, *n* = 7 in both treatments.

**Figure 3:**
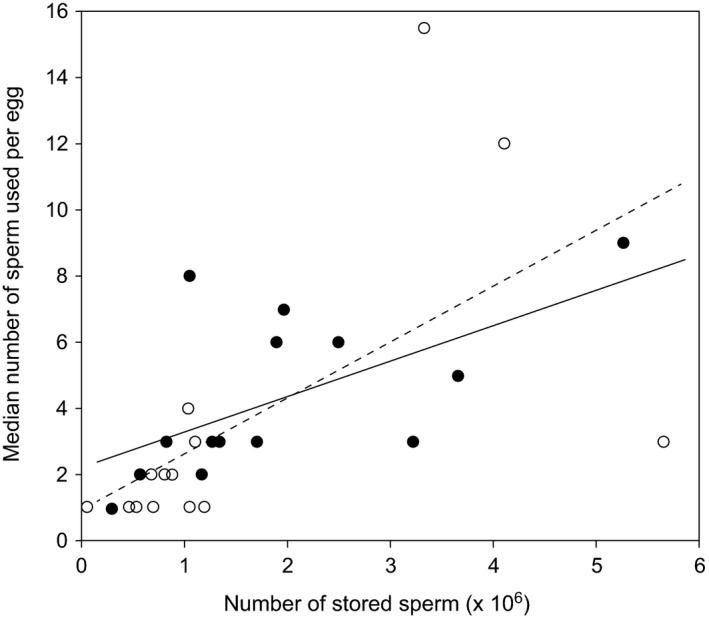
The relation between the number of sperm in the spermatheca and the number of sperm used per egg fertilization. The open symbols and dashed regression line (*y *= *βx* + *α*;* β* = 1.68 × 10^−6^, SE 6.32 × 10^−7^, *t* = 2.659, *P* = 0.0208; *α* = 0.938, SE 1.39, *t* = 0.679, *P* = 0.510; *N* = 14 queens) represent naturally mated queens of various ages (experiment 1). The closed symbols and line (*y *= *βx* + *α*;* β* = 1.11 × 10^−6^, SE 4.15 × 10^−7^, *t* = 2.665, *P* = 0.0206; *α* = 2.244, SE 0.962, *t* = 2.33, *P* = 0.0379; *N* = 14 queens) represent the artificially inseminated queens of similar age (experiment 2). The slope of each of these regression lines provides further estimates of *r*.

We found that queens inseminated with 12 *μ*L of ejaculate stored significantly more sperm in their spermatheca (2.725 × 10^6^ ± 5.492 × 10^5^, mean ± SEM) compared to queens inseminated with 3 *μ*L (1.098 × 10^6^ ± 2.269 × 10^5^; Mann–Whitney test, *U* = 6.000, *P* = 0.018; Fig 2). The number of sperm present in the spermatheca was a significant predictor for the number of sperm queens used for egg fertilizations; queens that store more sperm also used more sperm for egg fertilizations (*F*_1,12_ = 7.101, *P* = 0.021; Fig. 3). The same correlation between sperm usage and sperm number in storage was also found in the naturally mated queens used for the first experiment (*F*_1,12_ = 7.101, *P* = 0.021; Fig. 3).

### Sperm use economy of honeybee queens

**Figure 4:**
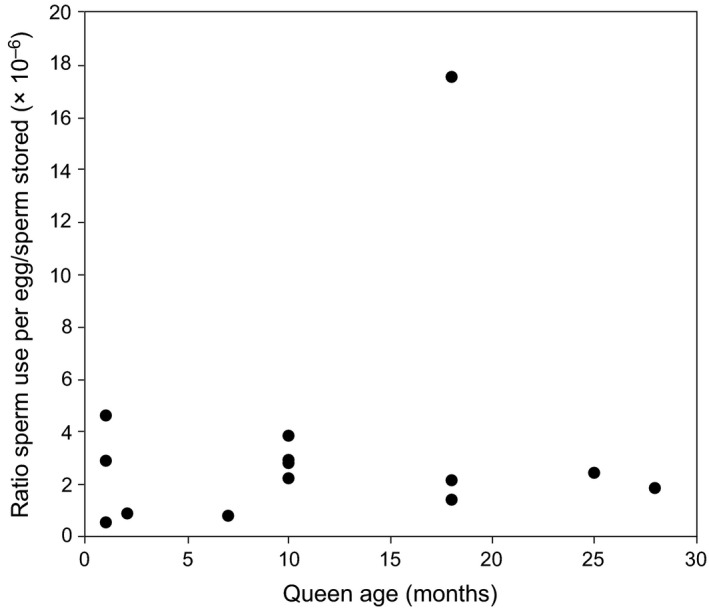
The relation between the age of the queen and the ratio of sperm use / sperm stored (*r*). The ratio is independent of the age of the queen (*F*_1,12_ = 0.448, *P* = 0.516).

The data obtained from naturally inseminated queens allowed us to analyze sperm use in honeybees in more detail. We first calculated the ratio of sperm used per egg to sperm stored (*r *= sperm used / sperm stored) for each queen (Median *r *=* *2.364 × 10^−6^, *N* = 14 queens). We did not find a significant relationship between *r* and age (*F*_1,12_ = 0.448, *P* = 0.516; Fig. 4), although the small sample means that the test has low power to detect anything but a strong relationship. An absence of a relationship led us to hypothesize that each queen uses the same volume of spermathecal fluid from the spermatheca for each fertilization, independently of the number of stored sperm or queen age. We also measured spermatheca width of these queens, allowing us to calculate the spermatheca volume *V* (Median *V *=* *0.8594 mm^3^). This allowed us to calculate the spermathecal fluid volume that each of the queens used to fertilize their eggs as F = V * r (median *F *=* *2.346 × 10^−6^ mm^3^, *N* = 14 queens). After calculating *r* values for the artificially inseminated queens of the second experiment we found no significant difference of *r* values between queens inseminated with 6 *μ*L and 12 *μ*L sperm and naturally mated queens (Kruskal–Wallis test, *H* = 5.161, *N* = 28, df = 2, *P* = 0.076).

### A model of sperm use

We developed a model of sperm use in honeybee queens in which a queen uses a constant volume of spermathecal fluid from the spermatheca for each fertilization. This allowed us to test whether the data we collected on sperm use in queens is consistent with this hypothesized mode of egg fertilization. It also offered us to calculate further variables such as the lifetime fecundity of a queen. Under this model, the number of sperm (*X*_*t*_) a queen uses for the insemination of egg number *t* depends on the sperm present in the spermatheca (*N*_*t*_), the volume of fluid used per fertilization (*F*), and the total volume of the spermatheca (*V*). For the fertilization of each egg, the queen uses:

(1) $$X_{t}=\frac{F}{V}{\left(1-\frac{F}{V}\right)}^{\left(t-1\right)}N_{0}$$

where *N*_0_ is the total number of sperm stored after mating (1)

This equation can also be expressed as a function of *r*:

(2) $$X_{t}=r{\left(1-r\right)}^{\left(t-1\right)}N_{0}$$

We can express equation (2) in linear form by taking the log of both sides. This then allows us to test this model by linear regression with sperm use data obtained for the naturally inseminated queens, as represented in Figure 1.

(3) $$\begin{array}{cc} \ln\left(X_{t}\right) & =\ln\left(r{\left(1-r\right)}^{\left(t-1\right)}N_{0}\right)\\ & =\left[\ln\left(r\right)+\ln\left(N_{0}\right)-\ln\left(1-r\right)\right]+\left[\ln\left(1-r\right)\right]t\\ & =A+Bt \end{array}$$

As *t* in equation (3) represents the number of the egg being fertilized, an assumption must be made as to the rate of egg fertilization. This does not affect the statistical significance of *r* in the regression, but does affect its value. If we assume 2000 eggs per day as the theoretically highest possible egg output of queens per day (Snodgrass 1984), we can obtain values for *r* and *N* using the 21 observations for naturally inseminated queens (*A *= ln(*N*_0_) + ln(*r*) − Ln(1 − *r*) = 1.67, SE 0.255, *t* = 6.527, *P* = 0.00183; *B *= ln(1 − *r*) = −1.11 × 10^−6^, SE 3.06 ×10^−7^, *t* = −3.617, *P* = 0.00183; *N* = 21 queens). Converting these to calculate *r* and *N*_0_, we get *r *=* *1.11 × 10^−6^ and *N*_0_ *=* 4,790,818.

The estimate of *r* is close to the median value of *r* calculated by examination of the volume of the spermatheca (*r *=* *2.364 × 10^−6^), whereas the value of *N*_0_ is close to other estimates of stored sperm numbers in the literature (such as the estimate of 4,730,000 in Koeniger and Koeniger 2000).

Using the derived values of *r* and *N*_0_, we are consequently able to describe sperm use of honeybee queens as:

(4) $$X_{t}=5.295\times 0.999998895^{t-1}$$

This equation matches the curve presented in Figure 1A, although the *y*‐axis denotes queen age in months in Figure 1A, rather than egg number. We can use equation (4) to calculate the number of eggs that queens are able to lay before they become infertile, which occurs when *X*_*t*_ = 1; that is, the concentration of sperm in the insemination sample drops below a single sperm. Resolving equation (4) for *t* reveals that a queen becomes infertile after laying 1,508,019 eggs.

Another estimate of *N*_0_ and *r* can be derived from the number of sperm in the spermatheca of naturally inseminated queens (as shown in Fig. 1B). The sperm stored in a queen as a function of age and *N*_0_ is:

$$N_{t}={\left(1-r\right)}^{t}N_{0}$$

Expressed in linear form by taking the log of each side, we can estimate *r* and *N*_0_ by linear regression:

$$\ln\left(N_{t}\right)=\ln N_{0}+\ln\left(1-r\right)t$$

If we again assume 2000 eggs per day, we can obtain values for *r* and *N*_*t*_ using the 14 observations for naturally inseminated queens (ln(*N*_0_) = 14.72, SE 0.380, *t* = 38.72, *P* = 5.67 × 10^−14^; ln(1 − *r*) = 1.39 × 10^−6^, SE 4.45 × 10^−7^, *t* = −3.126, *P* = 0.00875; *N* = 14 queens). Converting these to calculate *r* and *N*_0_, we get *r *=* *1.39 × 10^−6^ and *N*_0_ = 2,246,615. The curve of this regression is the same as that shown in Figure 1B.

## Discussion

We conducted a first direct quantification of sperm use in honeybee queens. As predicted, we found that queens are highly economic and use few sperm to fertilize eggs. We are not aware of other species, apart from the leafcutter ant *Atta colombica* (den Boer et al. 2009b), where females have such sperm use efficiency throughout their lives. This is remarkable given the astonishing fecundity of queens, both in terms of egg laying frequency as well as the total number of offspring they produce throughout their life. The mathematical model we developed to describe the economy of sperm use in honeybee queens was supported by our field observations. It provided a theoretical basis for the regressions reported in Figure 1A and B, as alternative models could also be developed. Furthermore, it confirmed that the data are consistent with the mechanism proposed, and allowed calculating of lifetime fecundities of queens, which were close to earlier estimates. Finally, the model allowed us to calculate novel parameters such as the average volume of secretion that queens release from the spermatheca for each individual egg fertilization.

Our finding of a median of 2 sperm per egg fertilization is lower than previously published estimates for honeybees, which ranged between 4 and 100 sperm per egg depending on the study and the methodology used (Bresslau 1905; Adam 1912; Ruttner 1975; Harbo 1979; Yu and Omholt 1999). This discrepancy is not surprising because we found sperm use of queens to be highly skewed and age dependent, and we therefore used biologically more relevant medians of sperm use rather than averages. Our data also provide detailed insights into factors affecting the sperm economy of honeybee queens. We found that the number of stored sperm was always the best predictor for the number of sperm observed on eggs, indicating that honeybee queens are unable to manipulate the number of sperm used during fertilization. The fertilization process of honeybee queens is best explained by a mechanism where a constant volume of sperm and spermathecal fluid is sampled from the spermatheca and then transferred onto the egg. As we found spermathecal size to be independent of queen age and sperm numbers stored, our findings imply that volumes taken out for fertilization are continuously replaced with spermathecal fluid. As the concentration of sperm decreases in the spermatheca, fewer sperm are used per fertilization for later eggs and our observed data nicely follow our mathematically derived model. This process of sperm use in honeybees has been hypothesized (Harbo 1979) but we here provide the first empirical evidence that this indeed is the case.

Honeybee queens possess a specialized morphological structure, known as the Bresslau sperm pump (Bresslau 1905) located between the spermatheca and the spermathecal duct. This structure consists of muscular tissue and a valve, which was hypothesized to be important for the transport of sperm to the spermatheca. We here provide an additional possible function of the Bresslau sperm pump as the morphological structure responsible for continuously sampling a constant volume of sperm and spermathecal fluid from the spermatheca and transferring it to the eggs. Such specialized parts in spermathecal ducts are not only known from honeybees, but are also present in ants (Oppelt and Heinze 2007). Consequently, such structures could provide the proximate mechanisms for the remarkable sperm economy in a number of eusocial insects, something that needs to be studied further in the future.

If queens are unable to manipulate their sperm use for female destined eggs, they cannot influence the fertilisation success of these eggs either. Consequently, we here provide empirical evidence for the presence of an honest signal for queen fertility in honeybees. As a queen lays fertilized eggs, the concentration of sperm decreases in the spermatheca to a point where she will start to lay nonfertilized eggs. It is then in her interest to get replaced by one of her daughter queens before she becomes completely infertile, as the latter will result in the death of the colony because virgin queens can only be reared from fertilized eggs. Consequently, our results imply no evidence for queen–worker conflict over supersedure in honeybees. What we predict from our data is that the appearance of haploid eggs in diploid comb could be a key factor initiating queen supersedure, something that can be experimentally tested in the future by transplanting haploid eggs into worker combs.

Our data on sperm use in honeybee queens is similar to those reported earlier for leafcutter ants, where queens also use an overall median of 2 sperm to fertilize their eggs (den Boer et al. 2009b). However, we found a marked difference in sperm economy between the two species, because sperm use in leafcutter ant queens increases with queen age, which was hypothesized to result from senescence of the queen's sexual tract and/or sperm. Sperm use of Atta queens increases only slowly over time though (den Boer et al. 2009b), suggesting that a phenotypic expression of senescence of sperm or the female's sexual tract requires years to manifest. Because the queens of honeybees live substantially shorter (years) compared to leafcutter ants (decades), especially in a Mediterranean climate such as in Western Australia, it is possible that such senescence effects do not become measureable in honeybees. Furthermore, leafcutter ant queens can initially store substantially more sperm in their spermatheca (465 million; Baer et al. 2006) and cannot be superseded. We can therefore conclude that although sperm economy has been driven to spectacular extremes in ants and bees, the proximate causes of achieving such spectacular levels of fertilization numbers and efficiency are based on different mechanisms. This is interesting, because sperm storage is an ancestral trait of hymenopteran social insects, and the storage of few sperm over shorter periods of time in ancestral social and nonsocial hymenopterans was then modified differentially and independently during the evolution of large and long‐lived insect societies. Obviously more research is required to understand the evolution of sperm economy in social insects, and termites might provide interesting opportunities for comparative approaches as they build societies of similar sizes and longevity, but the presence of males (kings) that continuously copulate with the queen should have eased selection on the efficiency of sperm storage and usage.

## Data accessibility

The data set that has formed the basis of the results presented here is available from the Dryad Digital Repository: http://dx.doi.org/10.5061/dryad.7nh16.

## Conflict of Interest

None declared.
